# The mistuning perception test: A new measurement instrument

**DOI:** 10.3758/s13428-019-01225-1

**Published:** 2019-03-28

**Authors:** Pauline Larrouy-Maestri, Peter M. C. Harrison, Daniel Müllensiefen

**Affiliations:** 1 Neurosciences Department, Max Planck Institute for Empirical Aesthetics, Frankfurt am Main, Germany; 2 School of Electronic Engineering and Computer Science, Queen Mary, University of London, London, UK; 3 Department of Psychology, Goldsmiths, University of London, London, UK

**Keywords:** Pitch perception, Musical abilities, Pitch accuracy, Gold-MSI

## Abstract

An important aspect of the perceived quality of vocal music is the degree to which the vocalist sings in tune. Although most listeners seem sensitive to vocal mistuning, little is known about the development of this perceptual ability or how it differs between listeners. Motivated by a lack of suitable preexisting measures, we introduce in this article an adaptive and ecologically valid test of mistuning perception ability. The stimulus material consisted of short excerpts (6 to 12 s in length) from pop music performances (obtained from MedleyDB; Bittner et al., 2014) for which the vocal track was pitch-shifted relative to the instrumental tracks. In a first experiment, 333 listeners were tested on a two-alternative forced choice task that tested discrimination between a pitch-shifted and an unaltered version of the same audio clip. Explanatory item response modeling was then used to calibrate an adaptive version of the test. A subsequent validation experiment applied this adaptive test to 66 participants with a broad range of musical expertise, producing evidence of the test’s reliability, convergent validity, and divergent validity. The test is ready to be deployed as an experimental tool and should make an important contribution to our understanding of the human ability to judge mistuning.

**Electronic supplementary material:**

The online version of this article (10.3758/s13428-019-01225-1) contains supplementary material, which is available to authorized users.

Mistuning in vocal performance is easily observed in everyday situations such as karaoke sessions, birthday celebrations, and sporting events. Individuals differ substantially in singing production abilities, and poor singing abilities often result in vocal mistuning, either through enlargement or compression of melodic intervals, or through misalignment with the instrumental accompaniment (Dalla Bella, Giguère, & Peretz, 2007; Hutchins & Peretz, 2012; Pfordresher & Brown, 2007; Pfordresher & Larrouy-Maestri, 2015). Whereas mistuning perception is an important musical ability, the research community lacks an effective tool for assessing it. As a result, little is known about how the ability is distributed in the general population, how it develops through adolescence, and how it relates to other musical abilities. To this end, this article introduces a quick and efficient test of mistuning perception abilities, focused on vocals, since this is a particularly salient component in the minds of listeners (Demetriou, Jansson, Kumar, & Bittner, 2018), termed the “Mistuning Perception Test.” We intend this test to be a useful tool for improving our understanding of how listeners perceive mistuning.

## Listeners’ mistuning perception ability

Lay listeners make reliable judges of vocal pitch accuracy in singing performances and have an intuitive notion of what sounds “correct” or “right” (Larrouy-Maestri, Magis, Grabenhorst, & Morsomme, 2015). This notion of correctness depends on musical rules learned implicitly through cultural exposure (e.g., Bigand & Poulin-Charronnat, 2006). Particularly relevant is the *tuning system*, which defines how the different degrees of the musical scale map to specific frequencies. In Western popular music, the tuning system is most commonly *equal temperament*, in which the musical scale is defined by dividing the frequency ratio 2:1 (termed the *octave*) into 12 equal ratios, each of size 2^1/12^:1 (Parncutt & Hair, 2018; for tuning systems in other musical styles, see Howard, 2007).

Various studies have investigated the perception of vocal mistuning (Hutchins, Roquet, & Peretz, 2012; Larrouy-Maestri, Lévêque, Schön, Giovanni, & Morsomme, 2013; Vurma & Ross, 2006; Warrier & Zatorre, 2002), but most of this work has come from the tradition of experimental psychology, with analyses typically marginalizing over participants. Conversely, individual differences in vocal mistuning perception remain relatively neglected. However, some recent work does suggest that mistuning perception is a stable ability with meaningful individual differences (Larrouy-Maestri, 2018). This study showed that listeners’ tolerance with regard to mistuning was stable over time and robust to different musical contexts, with listeners exhibiting large individual differences in ability (i.e., perceptual thresholds ranging from approximately 10 to 40 cents of intervallic deviation) that were positively predicted by musical expertise.

Little is known about the precise strategies used by listeners to detect vocal mistuning in everyday musical contexts, in particular when an instrumental accompaniment is present. One strategy would be to monitor the *dissonance* of the overall sonority; in Western music, mistuning tends to result in misaligned harmonic spectra, increasing roughness and decreasing harmonicity, thereby increasing perceptual dissonance (McDermott, Lehr, & Oxenham, 2010; Parncutt & Hair, 2011). Alternatively, the listener might separate out the vocal line from the accompaniment through auditory stream segregation (Bregman, 1990) and then assess the extent to which its pitch content conforms to the prototypical pitch distributions of the relevant musical style, or perhaps directly compare the vocal pitch to concurrent pitches in the instrumental part.

These strategies each place demands on different underlying musical abilities, such as dissonance perception, auditory stream segregation, and pitch discrimination. It is outside the scope of the present work to disentangle these different potential abilities. Instead, we begin with the simple approximation that mistuning perception is a unidimensional ability. However, if subsequent research identifies meaningful multidimensionality in mistuning sensitivity, this could be incorporated into future individual-differences tests.

## Testing mistuning perception ability

A long tradition exists of establishing listening test batteries for assessing musical talent or abilities, beginning with Seashore’s (1919) measures of musical talent. Some of these tools target the general population (i.e., Goldsmiths Musical Sophistication Index, Gold-MSI: Müllensiefen, Gingras, Musil, & Stewart, 2014; the Profile of Music Perception Skills, PROMS: Law & Zentner, 2012; the PSYCHOACOUSTICS toolbox: Soranzo & Grassi, 2014; the Swedish Musical Discrimination Test: Ullén, Mosing, Holm, Eriksson, & Madison, 2014; and the musical ear Test: Wallentin, Nielsen, Friis-Olivarius, Vuust, & Vuust, 2010); others target specialist populations, including musicians (e.g., the Test of Musical Abilities: Nikolić, 2017; or the Music Ear Training Assessment: Wolf & Kopiez, 2018), adults with hearing aids (Kirchberger & Russo, 2015; Uys & van Dijk, 2011), and amusics (Montreal Battery of Evaluation of Amusia: Peretz, Champod, & Hyde, 2003).

None of these preexisting batteries contains a test of sensitivity to vocal mistuning. Several tools exist, however, to examine mistuning perception with other types of stimuli. In the scale test of the PSYCHOACOUSTICS toolbox (Soranzo & Grassi, 2014), the listener is played an equal-tempered major scale beginning on C4 (261.6 Hz), synthesized as 500-ms complex tones in which the pitch of the fifth note (G4) varies by trial. The listener’s task is then to identify the mistuned scales. In the PROMS battery (Law & Zentner, 2012; see also the PROMS-Short and the Mini-PROMS, Zentner & Strauss, 2017), the listener is played a C major chord synthesized with a piano timbre and has to identify chords in which the major third (E4) has been mistuned, with the potential mistunings ranging from 10 to 50 cents.

These preexisting tasks are useful initial contributions, but they nonetheless have important limitations. Each only probes the perception of one tone (in the scale test of the PSYCHOACOUSTICS toolbox, the fifth scale degree; in the PROMS, the third scale degree), thereby leaving a large proportion of the potential musical material untested. The PSYCHOACOUSTICS test might also suffer from the fact that listener may focus on a single melodic interval and compare it to a mental representation of this specific (small) interval. A difficulty with the PROMS test is that the separation between comparison chords is very short, potentially allowing the listener to make direct pitch comparisons between the two versions of the relevant chord tone instead of judging mistuning. Moreover, the use of simple timbres (complex tones and piano tones) isolated from musical context makes these experimental tasks rather distant to real-life music listening, which often involves extracting pitch from complex timbres in a variety of musical textures and contexts. It seems likely that mistuning perception with realistic music may involve somewhat different skills to mistuning perception with simplified material, especially given the important role timbre seems to play in pitch perception (Micheyl, Delhommeau, Perrot, & Oxenham, 2006; Russo & Thompson, 2005; Vurma, Raju, & Kuuda, 2010), the established effect of preceding musical information of perceived mistuning (Warrier & Zatorre, 2002), and the vocal generosity effect described by Hutchins et al. (2012). We therefore believe that a mistuning perception test should preferably use realistic music material.

Finally, no previous mistuning tests have used the rigorous methodology of item response theory (IRT). IRT is the state-of-the-art approach to test construction, and has been recently used for assessing melodic discrimination ability (Harrison, Collins, & Müllensiefen, 2017; Harrison, Musil, & Müllensiefen, 2016), beat perception ability (Harrison & Müllensiefen, 2018), and ear training abilities in musically trained listeners (Wolf & Kopiez, 2018). IRT can also form the basis of adaptive testing procedures, which tailor their difficulty to the individual’s ability level through on-the-fly item selection. The combination of IRT and adaptive testing allows for substantial improvements in test efficiency, corresponding reductions in test length, and sophisticated estimates of measurement error (de Ayala, 2009; van der Linden & Glas, 2007).

## The development of a new test of mistuning perception ability

Our new mistuning perception test uses a two-alternative forced choice (2-AFC) paradigm in which each trial comprises two versions of the same musical extract, one of which is “in tune” and the other of which is “out of tune.” Each musical extract has a vocalist singing the main melodic line and an instrumental accompaniment. Out-of-tune extracts are produced by adding a constant pitch shift to the vocal line. The listener’s task is then to identify which of the pair was the out-of-tune version.

Our goal was to use musical extracts representative of the music normally listened to by Western individuals. To this end, we sourced our musical extracts from the MedleyDB dataset, which contains 122 popular music recordings of varying styles, performed in professional studios and mixed by experienced engineers, and available upon request for noncommercial purposes (Bittner et al., 2014; http://steinhardt.nyu.edu/marl/research/medleydb). This dataset also contains the individual tracks of the recordings, allowing each track to be analyzed and manipulated individually. We selected 37 short musical excerpts from this dataset following several criteria, including a natural singing style of the vocalist, popular and common musical genre (e.g., reggae, pop, rock), a clear tonal structure, a simple rhythmic structure, and a melodic line without any background chorus. We then used these materials to construct an IRT-based adaptive test. Our procedure followed the automatic item generation approach used in recent IRT-based adaptive musical tests (Harrison et al., 2017; Harrison & Müllensiefen, 2018). This approach can be split into three phases: *item construction*, *test calibration*, and *test validation*.

We began with *item construction*, in which we algorithmically generated a large number of potential test items. Here we used the short musical excerpts selected from the Medley database (see the supplementary information) to create 2,812 items representing mistunings, ranging from 10 cents to 100 cents, both sharp and flat. A large item bank is useful for adaptive tests, as it allows test difficulty to be precisely tailored to the ability level of the participant.

We continued with *test calibration*. The goal here was the quantitative estimation of each item’s difficulty. Under traditional IRT approaches (e.g., de Ayala, 2009), each item’s difficulty is estimated individually. Under the automatic item generation approach, item difficulties are estimated jointly through a statistical model predicting difficulty from structural item features (e.g., mistuning amount, musical style). One advantage of automatic item generation is improved calibration efficiency, meaning that fewer participants are required in the calibration phase (Harrison et al., 2017). A second advantage is the improved construct validity that comes from explicitly modeling different contributors to item difficulty (Harrison et al., 2017).

Test calibration enables the instantiation of the test’s final adaptive version. It is important to validate this test on new participants to verify the success of the design and calibration procedure. Here, we focused on two primary aspects of test quality: *reliability* and *construct validity*.

*Reliability* describes the consistency of test scores over repeated measurement. Reliability can be assessed by administering the same test multiple times to the same participant group, in what is called *test–retest reliability*. It is difficult to make principled comparisons of test–retest reliability between studies, because the standard metrics (Pearson correlation, intraclass correlation coefficient) do not generalize between participant populations and because test–retest reliability can be affected by practice effects, which can differ between tests (e.g., Bird, Papadopoulou, Ricciardelli, Rossor, & Cipolotti, 2003). Nonetheless, it is worth noting the mistuning test–retest reliabilities previously reported for the PROMS (*r* = .68; Law & Zentner, 2012), PROMS-Short (*r* = .47; Zentner & Strauss, 2017), and Mini-PROMS (*r* = .63; Zentner & Strauss, 2017). We are not aware of test–retest studies for the scale test of the PSYCHOACOUSTICS toolbox (Soranzo & Grassi, 2014), but the test–retest reliability for the pitch discrimination test of this toolbox is high (*r* = .87; Smith, Bartholomew, Burnham, Tillmann, & Cirulli, 2017).

*Construct validity* describes the extent to which the test measures the theoretical construct it is intended to measure. Construct validity is difficult to assess definitively but a typical approach is to collect information on how test scores correlate with other test scores. This concept has been termed *nomothetic span* (Embretson, 1983). When high correlations are observed that are consistent with psychological theory, this is termed *convergent validity*. Conversely, low correlations consistent with psychological theory are evidence for *divergent validity*. Previous research with the PROMS (Law & Zentner, 2012) found tuning perception to correlate well with a variety of other musical traits, as assessed by the eight other PROMS subtests (coefficients ranging from .47 to .71; Law & Zentner, 2012), by non-PROMS tests (e.g., *r* = .48 with the Advanced Measures of Music Audiation [AMMA] tonal test; *r* = .41 with the AMMA rhythm test; and *r* = .28 with the MET rhythm test; Law & Zentner, 2012), and years of musical training, musicianship status, self-rated musical talent, and harmonic closure judgment (Kunert, Willems, & Hagoort, 2016). Conversely, PROMS tuning performance has been shown not to correlate with ostensibly nonmusical skills such as gap detection (Law & Zentner, 2012; despite the fact that gap detection seems to be a good discriminator of musicians from nonmusicians—see, e.g., Grassi, Meneghetti, Toffalini, & Borella, 2017) and digit span (Kunert et al., 2016). These results might be summarized by saying that tuning perception ability seems to be associated with a variety of musical skills—including, perhaps surprisingly, skills not obviously related to pitch perception—but there is little evidence for associations with nonmusical skills. This prior research provides the context for assessing the construct validity of our new test.

To summarize, we constructed a new mistuning perception test with the following goals: to use realistic stimuli, to give precise ability estimates while keeping test length short (less than 10 min), and to be easily understood by children and adults without formal musical training. In addition to improving test reliability and construct validity, we aimed to create an enjoyable testing experience for the participant, making the test well-suited for future large-scale testing.

## Experiment 1: Test construction and calibration

### Method

The Ethics Council of the Max Planck Society and the Ethics Board at Goldsmiths, University of London, approved both experiments. Informed consent was obtained from all participants tested in the laboratory.

#### Participants

The participant group numbered 333 individuals (227 women, 106 men), with ages ranging from 18 to 70 years (*M* = 24.76, *SD* = 7.95). Of these, 185 participated in the laboratory, and the remaining 148 participated online. The group exhibited large variation in musical training, with Gold-MSI musical training scores ranging from 7 to 48 (*M* = 24.21, *SD* = 10.94) (Müllensiefen et al., 2014; theoretical range from 7 to 49).

#### Material

##### Selection of the material

A total of 37 short musical excerpts were selected from the MedleyDB dataset (Bittner et al., 2014). This royalty-free dataset contains stereo mixes as well as preprocessed multitracks and postprocessed stems for each song. The available material (*n* = 122) represents popular music genres such as pop, reggae, rock, gospel, music theatre, and country. The instrumentation of these songs is dominated by drums, bass, piano, guitar, and vocals. As was reported in Bittner et al. (2014), most songs were recorded in professional studios and mixed by experienced engineers.

The selected 37 excerpts contained musical material from 22 composers/bands. For the seven composers/bands that appeared multiple times, their selected contributions were chosen to represent contrasting musical genres (see the supplementary information). Only material containing solo vocals as the melodic line were considered, with 20 excerpts from male singers and 17 from female singers. The selected excerpts were restricted to cases in which the melodic line was performed with a natural singing style, thereby excluding idiosyncratic vocal styles such as are common in operatic music and metal music.

Excerpts were chosen to stand as independent units. Each excerpt comprised one melodic phrase, with clear tonal and rhythmic structure, corresponding to a well-defined linguistic sequence such as a phrase or a section of repeated words/syllables. As a consequence of this constraint, the excerpts varied markedly in length, with length ranging from 4.91 to 14.4 s (*M* = 9.07, *SD* = 2.33). Shorter lengths were avoided, so as to provide sufficient information for mistuning judgments; longer lengths were avoided so as to minimize demands on working memory and to keep overall test length within reasonable limits. As a result of the variety in genre and length, the excerpts also varied substantially in melodic pitch range (1 to 16 semitones; *M* = 8.81, *SD* = 3.35), number of syllables (2 to 34; *M* = 14.50, *SD* = 6.85), number of tones (4 to 30; *M* = 14.81, *SD* = 6.32), and tempo (60 to 132 beats per minute; *M* = 93.41, *SD* = 24.52). Excerpts were extracted from the original audio files using Adobe Audition (https://www.adobe.com/products/audition.html), and 500-ms fade-ins/-outs were applied to the beginnings and ends of the extracts.

##### Manipulation of the material

Vocal tracks were pitch-shifted using the software SoX (http://sox.sourceforge.net/). In each case, the pitch shift was constant for the entire duration of the extract (i.e., no random variation). Pitch shifts were performed in both directions (flat and sharp) and ranged from 10 to 100 cents in 5-cent increments. Smaller mistuning deviations than 10 cents were not considered, since it was difficult to guarantee that these mistunings would exceed preexisting mistuning deviations in the original musical tracks. Pitch-shifted vocal tracks were then combined with the other (in-tune) tracks to produce the final mix using SoX.

##### Presentation of the material

In total, a set of 2,812 stimuli was created, each corresponding to a 2-AFC item. Each 2-AFC item comprised the original (i.e., in tune) and a mistuned version of a musical excerpt, separated by 1 s of silence. These items were created by the factorial manipulation of four variables: musical excerpt (37 levels), amount of pitch shift (19 levels: ranging from 10 to 100 cents in 5-cent increments), direction of pitch shift (two levels: sharp or flat), and position of the mistuned version (two levels: first or second). The resulting stimulus set is available here: https://edmond.mpdl.mpg.de (collectionMistuningPerceptionTest).

#### Procedure

For both the online and lab sessions, testing was conducted using the online platform “Concerto” (Scalise & Allen, 2015). In the lab, stimuli were presented via headphones (either K271 MKII [AKG, Vienna, Austria] or HPM1000 [Behringer, Germany]) at a fixed comfortable loudness level (about 65 dB) in a group testing room or in a single sound attenuated booth. For the online sessions, participants were strongly advised to wear headphones and to take the test in a quiet room free from interruptions.

Prior to the data collection phase, the mistuning test began with a training phase, including instructions, audio demonstrations, and two practice trials. Participants were then presented with 37 stimuli from the stimulus set, chosen randomly with the constraint that no participant heard the same musical excerpt twice. In each case, they were instructed to determine whether the “out of tune” excerpt came first or second. No feedback was provided for these trials.

After completing the mistuning test, participants then took the Gold-MSI self-report questionnaire (Müllensiefen et al., 2014), including a short questionnaire concerning their age, gender, and occupational status.

#### Analysis

The analysis comprised two steps. First, the musical excerpts were screened for inclusion in the future test. The criterion we used was the relationship between item difficulty and magnitude of the pitch shift. We reasoned that, for a “well-behaved” musical extract, mistuning perception difficulty should be strongly influenced by the amount of pitch shift, with larger shifts corresponding to easier items (e.g., Larrouy-Maestri et al., 2015). Concretely, we examined the Pearson correlations between mean success rate and pitch shift level across all musical excerpts, as well as at the level of individual musical excerpts. Second, we calibrated the item bank, modeling item difficulty using an explanatory item response model that predicted item responses from a linear combination of item features (e.g., mistuning amount) and person features (e.g., musical training).

### Results and discussion

#### Selection of musical excerpts

**Fig. 1:**
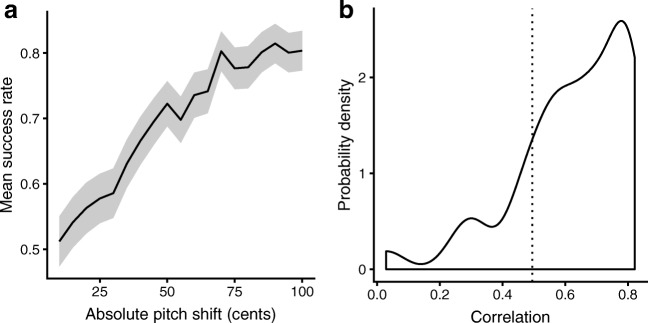
Relation between listeners’ performance and mistuning. (A) Mean success rate as a function of absolute mistuning (the shaded confidence ribbon represents the 95% confidence region), calculated for the 37 original musical excerpts from the MedleyDB (Bittner et al., 2014). (B) Kernel density plot illustrating the distribution of Pearson correlation coefficients between pitch shift and mean success rate at the level of individual excerpts. The fitted line estimates the probability density function for the hypothetical population from which the musical excerpts were sampled. The excerpts (*n* = 7) with correlations less than *r* = .495 (dotted line) were discarded

Figure 1A plots amount of pitch shift against the mean success rate, averaged across all musical excerpts. The response data come from 333 participants, 185 of which participated in person, and 148 of which participated online. The trend is close to linear [Pearson *r*(17) = .965, *p* < .001] and covers success rates from 51.2% to 81.5% (where chance level corresponds to 50%).

Figure 1B describes the relation between pitch shift and mean success rate at the level of individual musical excerpts. The excerpt-wise correlations are lower than the aggregated correlation; this is likely due in part to statistical noise in estimating success rates, and in part to mistuning inaccuracies and ambiguities. The correlations vary by excerpt (minimum *r* = .029, maximum *r* = .822; *M* = .625, *SD* = .185), indicating that it should be possible to raise the overall quality of the excerpts by excluding tracks with low correlations. We decided to exclude the seven musical excerpts with correlations lower than .495 (*r* = .5 being considered a large effect size for correlational studies; Cohen, 1988), thus keeping 30 excerpts from the initial set (in the supplementary information).

#### Modeling item difficulty

We then proceeded to calibrate the item bank. This involved constructing a statistical model capable of predicting each item’s IRT parameters, in particular its difficulty, on the basis of its structural item features. We estimated IRT parameters using explanatory item response modeling (de Boeck & Wilson, 2004), allowing us to use a cognitive–statistical model of task performance to improve the efficiency of parameter estimation. The item response model took the form of a generalized linear mixed-effect model with a logit link function (also known as *mixed-effect logistic regression*) and modified asymptotes (Harrison & Müllensiefen, 2018). This model reproduces a four-parameter logistic IRT model in which the guessing, discrimination, and inattention parameters are constrained not to vary within the item bank (Magis & Raîche, 2012). In addition to calibrating the item bank, this approach has the function of quantifying the influences of different item features on difficulty, as well as the relationship between participant features and ability, both of which are useful for the test’s construct validity.

We tested three item effects and one person effect, each representing hypotheses about how item features determine difficulty and how person features determine ability. The item effects were the amount of pitch shift (a continuous variable), the direction of pitch shift (a binary variable), and the musical excerpt (a categorical variable). The person effect was self-reported musical training (a continuous variable). All continuous variables were scaled to *z* scores. Floor and ceiling success rates (under IRT terminology, guessing and inattention parameters) were initially constrained to 50% and 100%, respectively.

Specifying the random-effect structure of the generalized linear mixed-effect models was a difficult balance between properly accounting for the hierarchical nature of the data and ensuring convergence of the statistical model estimation process. We constructed a range of seven candidate models with different fixed-/random-effect structures and applied them to response data from the 333 participants. Following the model selection strategy outlined in Long (2012) we selected the best of the converging models using the corrected Akaike information criterion (AICc) measure, which balances model fit with parsimony. The ΔAICc between the best and second-best models was 2, and the best model had an AICc weight of 0.48, indicating a substantial effect size. Having identified an optimal effect structure, we then optimized the ceiling success rate (i.e., inattention) parameter using maximum-likelihood estimation.

As is reported in Table 1, the best model had pitch shift amount and musical training as fixed effects, participant and musical excerpt as random intercepts, and pitch shift amount and direction as random slopes with musical excerpt. From the fixed effects, it can be inferred that greater pitch shift and greater musical training both increased the probability of a correct response. From the random effects, it can be inferred that participants differed in mistuning perception ability, that musical tracks varied in their difficulty, that the relationship between pitch shift and difficulty was moderated by musical track, and that pitch shifting in a particular direction made some items harder but other items easier. These effects are consistent with the assumption that small tuning deviations in the original musical excerpts exist and might differ from one excerpt to the other.^1^ The ceiling success rate was estimated at 98.3%.

**Table 1:** Final model parameters

Effect	Type	Estimate (Standardized)	*SE*
Intercept	Fixed	– 0.432	0.198
Pitch shift amount	Fixed	1.294	0.061
Musical training	Fixed	0.667	0.080
Participant	Random intercept	1.136	NA
Mistuning direction : Song	Random slope	1.088	NA
Song	Random intercept	0.961	NA

Random effect estimates are reported as standard deviations. NA, not applicable.

In the case of solo melodies, it has been observed that the enlargement or compression of intervals between consecutive tones size is the best predictor for pitch accuracy judgments (Larrouy-Maestri et al., 2015) and that tolerance threshold is highly consistent whatever the direction of the deviation (enlargement or compression), or the position or size of the interval manipulated within the melody (Larrouy-Maestri, 2018). The consistency of tolerance with regard to mistuning in the case of pop music remains to be explored but our results already confirm that the absolute pitch shifts can be considered as a relevant criterion for the Mistuning Perception Test.

The model was then used to generate IRT parameters for the item bank. The resulting item difficulty parameters incorporated both the fixed and random effects from the calibrated item response model. As is conventional in IRT, the parameters were scaled so that a distance of one unit on the difficulty scale corresponded to the standard deviation of participant ability in the sample group. These parameters formed the basis of the subsequent adaptive test version.

## Experiment 2: Test validation

### Method

#### Participants

Sixty-six participants (40 women, 26 men) took part in the validation experiment, of which 62 completed the test and the retest within 10 days (*M* = 4.42 days, *SD* = 2.34) and one completed the retest 19 days after the test. The participants’ ages ranged from 19 to 34 years (*M* = 24.32, *SD* = 3.88). All participants were administered the adaptive mistuning test as well as six further tasks to examine the convergent and divergent validity of the test.

#### Material and procedure of the adaptive mistuning test

The adaptive mistuning test used the item bank described in the first experiment (calibration phase) minus the seven excluded excerpts*.* The final set thus included 30 excerpts (see the supplementary material) and an item bank of 2,280 items. Item selection was guided by Bayes modal ability estimation, with ability estimates being recalculated after each participant’s response. Each successive item was selected using Urry’s rule (Magis & Raîche, 2012), under the constraint that no participant heard the same musical excerpt twice. Final abilities were estimated using weighted likelihood estimation (Warm, 1989).

Before taking the adaptive test, participants took a training phase comprising instructions, audio demonstrations, and two practice questions. The stimuli were presented via headphones (K271 MKII [AKG, Vienna, Austria]) on the platform Concerto (Scalise & Allen, 2015), at a fixed comfortable loudness level (about 65 dB), in a single sound-attenuated booth. As with the calibration phase of the first experiment, participants were instructed to determine whether the out-of-tune excerpt came first or second, and they did not receive any feedback.

#### Comparison measures for convergent and divergent validity

##### Gold-MSI self-report questionnaire (Müllensiefen et al., 2014)

This short questionnaire (~ 7 min) addresses various aspects of musical behavior and expertise. It comprises five subscales (Active Engagement, Emotions, Musical Training, Perceptual Abilities, and Singing Abilities) and a general factor drawing on all subscales. Data from a large sample (*n* = 147,663) of the general population (Müllensiefen et al., 2014) indicated a wide range of general musical sophistication scores, ranging from 32 to 126 (*Mdn* = 82, *M* = 81.52, *SD* = 20.62) as well as a large variability in formal musical training, ranging from 7 to 49 (*Mdn* = 27, *M* = 26.52, *SD* = 11.44).

##### Duration discrimination (Grassi & Soranzo, 2009)

This test assesses a listener’s duration discrimination threshold, or the minimum duration difference that the listener can detect between two stimuli. The test uses a three-alternative forced choice (3-AFC) paradigm; in each trial, listeners are presented with three complex tones in quick succession (four harmonics, fundamental frequency = 330 Hz), two of which are 250 ms in length, and one of which is somewhat longer (starting length: 450 ms). The listener’s task is determine the longer tone. Over the course of the test, the duration difference between tones adapts to the listener’s performance using the maximum-likelihood procedure, and ideally converges on the listener’s duration discrimination threshold. Each participant’s duration discrimination threshold was estimated by running the adaptive procedure six times, with 25 trials per procedure, and computing the average of the six threshold estimates. These six repetitions were separated by short breaks. To the best of our knowledge, no norms have been published for this test, but in the validation phase we observed thresholds ranging from 18.28 to 89.38 ms (*M* = 38.40, *SD* = 13.71), with the total task lasting about 10 min.

##### Pitch discrimination of complex tones (Grassi & Soranzo, 2009)

This test assesses a listener’s pitch discrimination threshold, the minimum frequency difference that they can detect between two pitches. The test uses a 3-AFC paradigm; in each trial, listeners are presented with three complex tones in quick succession (four harmonics, 250 ms per tone), two of which have fundamental frequencies of 330 Hz, and one of which has a somewhat higher frequency (starting frequency: 390.01 Hz). The listener’s task is to determine the highest tone. Analogously to the duration discrimination task, thresholds were estimated using a maximum-likelihood procedure with six repetitions, 25 trials per procedure, short breaks between procedures, and with a total task duration of about 10 min. We are not aware of published norms for the exact same task and material (i.e., 3-AFC with complex tones), but Micheyl et al. (2006) observed pitch discrimination thresholds of about 15 cents in non-musically-trained listeners and 2 cents in formally trained listeners (with a different task using complex tones), whereas Smith et al. (2017) observed a median threshold of about 14.50 cents (same task using pure tones). In our experiment, pitch discrimination thresholds ranged from 6 to 116 cents (*M* = 35.30, *Mdn* = 24.00, *SD* = 29.62).

##### Beat perception (Harrison & Müllensiefen, 2018)

Beat perception ability was assessed using the computerized adaptive beat alignment test (CA-BAT) of Harrison and Müllensiefen (2018). This test uses a 2-AFC task in which each item comprises two versions of a musical excerpt, both superimposed with a metronome-like series of tones termed the *beep track*. In one version the beep track is fully aligned with the musical beat; in the other version the beep track is displaced ahead of the beat or behind the beat. Participants have to determine which version is correctly aligned. The test is intended to assess the participant’s ability to infer the beat in a musical piece. We used the full 25-item test with identical psychometric parameters and adaptive procedure to the original study (Harrison & Müllensiefen, 2018).

##### Melodic discrimination abilities (Harrison, Collins, & Müllensiefen, 2017)

Melodic discrimination ability was assessed using the adaptive melodic discrimination test of Harrison et al. (2017). This test uses a 3-AFC task in which each item comprises three versions of a melody at different transpositions. Two of these versions are always identical (ignoring transposition), and one is always different. The participant’s task is to identify the nonidentical melody. We used the full 20-item test with identical psychometric parameters and adaptive procedure to the original study (Harrison et al., 2017).

##### Tuning test from the Profile of Music Perception Skills battery (PROMS; Law & Zentner, 2012)

This test consists of pairs of C major chords (C4, E4, G4), each 1.5 s in length, synthesized with a piano timbre. Each listener is presented with 18 such pairs. In half of these pairs, the two chords are tuned correctly according to equal temperament; in the other half, the middle tone of one chord of the pair (E4) is frequency-shifted by an amount ranging from 10 to 50 cents. For each pair, the participant is asked to judge whether the two chords are the same or different. The possible raw scores range from 0 to 18. In our sample, the scores ranged from 4.5 to 15.5 (*M* = 10.18, *SD* = 2.47). These values are close to the values reported by the original authors for a subsample of *n* = 36 from which professional and semiprofessional musicians were removed (*M* = 11.74, *SD* = 2.48).

### Results and discussion

#### Reliability

Having administered the final version of the adaptive test to a new participant group, reliability was assessed by means of two measures: test–retest reliability and IRT standard error. Test–retest reliability describes the consistency of test scores over repeated testing, and is measured here as the Pearson correlation between ability estimates from a test session and a retest session. Unlike test–retest reliability, IRT standard errors have the advantage that they can be computed from a single test session. However, they rely more on model assumptions than do most test–retest reliability measures.

**Fig. 2:**
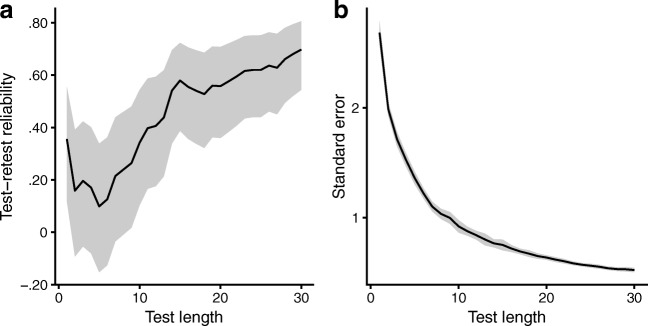
Reliability as a function of test length. (A) Evolution of test–retest reliability (Pearson correlation) as a function of test length (*n* = 30 items). (B) Mean standard errors (*SE*s) of the mistuning perception ability estimates, plotted as a function of test length. For panels A and B, the shaded ribbon represents the 95% confidence region

Test–retest reliabilities and IRT standard errors are plotted in Fig. 2 as a function of test length. As might be expected, reliability increased with longer test lengths, peaking at 30 items, with a test–retest reliability of .698 (95% CI: [.544, .806]; Fig. 2A) and a mean standard error of .521 (Fig. 2B). However, Fig. 2 also suggests that shortened versions of the test might also be possible; the increase in test–retest reliability seems to slow down somewhat after 15 items (*r* = .579, 95% CI: [.387, .723]), so a 15-item test might be practical when time is limited.

#### Construct validity and nomothetic span

**Fig. 3:**
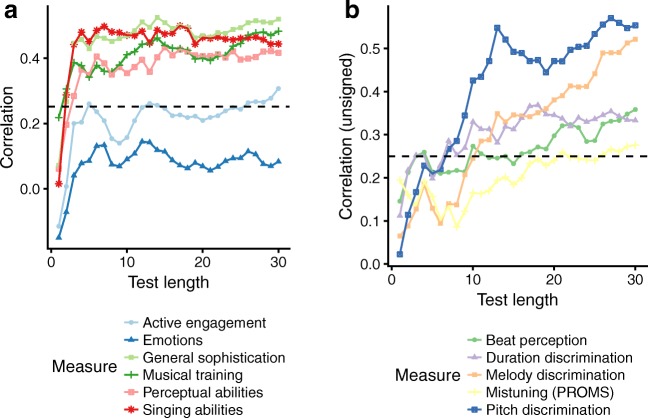
Correlations between adaptive mistuning perception test scores and other measures as a function of test length. (A) Correlations with the six questionnaire-based subscales of the Gold-MSI (Müllensiefen et al., 2014). (B) Correlations with two pitch-based tasks (pitch and melody discrimination), two temporal tasks (duration discrimination and beat perception), and the tuning task from the PROMS (Law & Zentner, 2012). The dotted lines represent the threshold for two-tailed statistical significance at a level of *p* < .05

To understand precisely what traits are measured by a test, it is useful to investigate how test scores correlate with other preestablished trait measures (Embretson, 1983). High correlations suggest that the two instruments measure related psychological traits, whereas low correlations suggest distant psychological traits. Here we investigated nomothetic span by correlating tuning discrimination scores with scores from 11 different measures of musical ability (Fig. 3). Six of these measures were questionnaire-based, and five were listening tests. Plotting the correlations as a function of test length provides information about the potential for shorter test lengths.

Correlations with the questionnaire-based measures are plotted in Fig. 3A. These measures correspond to the six subscales of the Gold-MSI (Müllensiefen et al., 2014), and assess different aspects of musical experience, behavior, and self-perceived abilities. Moderate correlations were found for musical training, perceptual abilities, singing abilities, and general musical sophistication. Low or nonsignificant correlations were found for the two remaining aspects: active engagement and emotion. Interestingly, these correlations stabilized very quickly, plateauing at approximately *r* = .4 after only four items.

Correlations with the five listening measures are plotted in Fig. 3B. The highest correlations were with pitch discrimination and melody discrimination abilities; pitch discrimination is a low-level task of detecting small frequency differences between tones, whereas melody discrimination is a high-level task of detecting changes in pitch content between melodies. However, correlations with the mistuning test from the PROMS barely reached statistical significance, even after 30 items. Moderate correlations were found with two tests of temporal abilities: duration discrimination and beat perception.

#### Distributions of person abilities and item difficulties

**Fig. 4:**
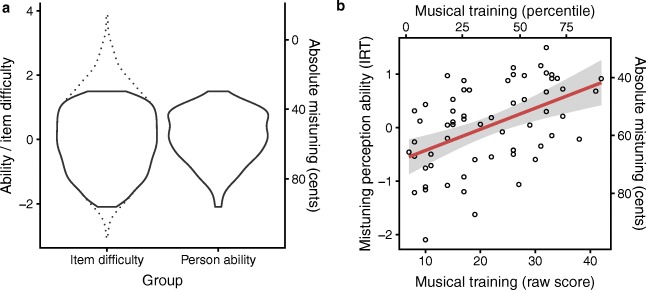
Distributions of person abilities and item difficulties for the adaptive mistuning perception test. (A) Kernel density estimates of person ability distributions versus item parameter distributions. Ability/difficulty parameters are given on an IRT metric (*z* scale, left-hand *y*-axis) and on a perceptual-threshold metric (in cents, right-hand *y*-axis). The right-hand *y*-axis (in cents) uses the item response model to map item difficulty to absolute mistuning amount for an “average” musical track. Note that this mapping gradually loses reliability when extrapolating outside the observed person ability range (dotted lines), and in extreme cases delivers paradoxical results (e.g., mapping an item difficulty of 4 to a negative absolute mistuning). (B) Test performances as a function of musical training scores from the Gold-MSI self-report questionnaire (Müllensiefen et al., 2014). A least-squares regression line is plotted, with the standard error of its predictions shaded in gray. Ability/difficulty parameters are given on both an IRT metric and a perceptual-threshold metric. Musical training is represented by raw scores and by percentile scores with reference to the original Gold-MSI study (Müllensiefen et al., 2014)

A useful feature of IRT is that item difficulties and person abilities are defined on the same metric, a *z*-score scale typically ranging from – 4 to 4. This means that, for example, a participant with an ability score of 1 is 1 *SD* above the population mean, and an item with a difficulty of 1 would be well-suited to a participant with ability 1. Figure 4A plots the distribution of item difficulties of all 2,280 items in the item databank as derived from the explanatory IRT model, alongside the distribution of participant abilities as measured by the adaptive version of the mistuning test. The difficulty distribution completely covers the ability distribution, which is a good sign: it means that the adaptive test can effectively tailor its difficulty to both the worst and the best participants.

Item difficulty can be linked to item features through the explanatory item response model. The biggest predictor of item difficulty is absolute pitch shift (despite any natural intonation variability in the original versions that might have introduced statistical noise), so Fig. 4A also represents difficulty in terms of absolute pitch shift (marginalizing over the other difficulty predictors). In all, 95% of participant abilities fell in the range [– 1.369, 1.136] (*Mdn* = 0.115), corresponding to a pitch shift range of [36.309, 82.589] (*Mdn* = 54.989) cents. In contrast, the corresponding 95% interval for the pitch discrimination task was [8.0, 111.8] (*Mdn* = 24.0) cents.

Without further testing, the sample distribution in Fig. 4A has limited utility as a norm, because our participant group is unlikely to be truly representative of the general population. However, better norms can be achieved by conditioning the ability distribution on musical training. We know that musical training is a key predictor of mistuning perception ability (Fig. 3A), so conditioning on musical training should account for a good proportion of background differences between potential sample groups. Figure 4B provides this information, plotting a least-squares linear regression line that provides norms for mistuning perception ability at different levels of musical training. For example, a low musical training raw score of 10 on the Gold-MSI training subscale corresponds to a mistuning perception threshold of 65.173 cents, whereas a high raw score of 40 corresponds to a threshold of 43.219 cents. The researcher can use this information to determine whether a particular participant has high or low mistuning perception ability, given their musical training background.

## Conclusion

The new Mistuning Perception Test provides an efficient way for researchers to quantify a listener’s ability to evaluate intonation accuracy in musical performance. Two properties in particular distinguish it from previous tests: the use of ecologically valid musical stimuli, representative of real-life music, and the application of modern psychometric techniques, including IRT, adaptive testing, and automatic item generation.

Our validation analyses suggest that the test possesses the reliability and validity to be deployed as a useful research tool. Good reliability—that is, high measurement precision—is indicated by the high test–retest reliability and the low standard error of measurement as derived from the IRT model. Good construct validity is provided by the collection of pairwise correlations with measures of different musical and auditory abilities.

The network of correlations with musical traits deserves further discussion. The widespread positive correlations with other musical traits support the notion that mistuning perception is a core musical ability, as suggested in previous literature (Kunert et al., 2016; Law & Zentner, 2012). In particular, the high correlation with self-reported singing abilities is interesting given prior work linking vocal production to vocal perception abilities (e.g., Hutchins, Larrouy-Maestri, & Peretz, 2014; Maes, Leman, Palmer, & Wanderley, 2014) as well as the known difficulty of accurately self-estimating singing abilities (Pfordresher & Brown, 2007) and the dissociation between perception and production reported elsewhere (Zarate, Delhommeau, Wood, & Zatorre, 2010). It would be worth replicating this analysis with an objective (i.e., not self-reported) measure of singing ability. Lower correlations were found with self-reported active engagement and emotional responsivity to music; this might suggest that intonation perception is not closely connected to the emotional aspects of aesthetic experience (Leder, Belke, Oeberst, & Augustin, 2004).

High correlations were found with two pitch-related ability tests: pitch discrimination (a low-level pitch-processing ability) and melody discrimination (a high-level pitch-processing ability). This supports the idea that pitch processing is a key part of mistuning perception. Conversely, correlations with the temporal processing tests (beat perception and duration discrimination) were relatively low, confirming a relative independence between duration discrimination abilities and other musical abilities and activities (Grassi et al., 2017; Smith et al., 2017). This is consistent with theoretical models of music processing that describe a dissociation between pitch and time in music perception (Peretz & Coltheart, 2003; such models have been highly discussed since then—e.g., by Prince, 2011).

Interestingly, the observed correlation between our test and the PROMS tuning subtest was rather low. Given the documented reliability of the PROMS tuning subtest (Kunert et al., 2016; Law & Zentner, 2012), this low correlation suggests that the two tests measure fundamentally different abilities. As we discussed previously, the PROMS subtest uses simple stimuli (major triads played by synthesized piano) and presents comparison chords with little temporal separation, allowing direct comparison between the absolute pitches of tuned and mistuned tones in echoic memory. In contrast, our stimuli are considerably more complex, varied, and representative of real music. Moreover, our stimuli were relatively long, making it difficult for listeners to perform the task solely using direct comparison, since the temporal separation between repetitions of particular musical elements (which is equal to extract length plus silent gap duration) likely exceeds the span of echoic memory.

The new test’s item bank covers a wide spectrum of mistuning perception abilities. This should make it appropriate for assessing listeners of varying ages and musical training levels, and indeed we have recently applied the test to a large group of children (circa 750) with ages ranging between 10 and 18 years (for study design see Müllensiefen, Harrison, Caprini, & Fancourt, 2015). Early results suggest that the test can be used successfully with children as young as 10. A useful property for large-scale adoption of the test is the engagingness that derives from the use of varied and realistic music (as opposed to repetitive major scales or triads), from the way in which the adaptive procedure ensures that the participant is always challenged but not challenged too much, and from the shorter test lengths made possible by the adaptive procedure. Further motivation could be elicited by providing the participant with instant feedback of their score at the end of the test.

The adaptive procedure makes the test efficient and relatively short (about 10 min with 30 trials). However, analyzing reliability and validity as a function of test length suggests that the test could be shortened to around 15 items without losing much of its psychometric strengths. The test is freely available (doi:10.5281/zenodo.1415363), in an implementation suitable either for laboratory testing or online testing. In online testing, an important consideration is the lessened control over environmental conditions and quality of sound presentation. However, these problems may be mitigated to some extent by recent psychophysical tests that ensure that participants use headphones (Woods, Siegel, Traer, & McDermott, 2017).

Although the present test seems already suitable for research applications, additional benefits could be achieved through further test development. Test reliability could be further increased by improving the statistical model of item difficulty, perhaps by allowing for nonlinearity in the relationship between mistuning and item difficulty, by better modeling individual differences between different musical excerpts, or by taking into account other features that might affect mistuning perception, such as scoops (i.e., pitch variations at the start and end of tones; Larrouy-Maestri & Pfordresher, 2018). The generalizability of the test could be improved by introducing different kinds of mistunings, in particular nonsystematic pitch error as opposed to systematic pitch shift, and by introducing nonvocal music.

The mistuning perception test’s efficiency is fundamentally limited by the 2-AFC paradigm, which requires the participant to be played two musical extracts to collect one “bit” of information (“correct” or “incorrect”). An interesting possibility would be to reimplement this test using the “method of adjustment,” in which the listener continuously manipulates the pitch shift of the vocal line until he or she believes it to be completely in tune (e.g., with a slider, as was used in Hutchins et al., 2014). Each trial of the test would then deliver considerably more information (i.e., the precise accuracy of the participant’s adjustment). However, the method of adjustment brings its own complications, since it is sensitive to the participant’s strategy and the particular experimental interface used (Wier, Jesteadt, & Green, 1976).

The new mistuning perception test should prove useful for tackling fundamental questions about the nature of musical ability. The correlations reported in the present article already provide useful information about potential associations between mistuning perception and other musical abilities, such as singing ability, and pitch-processing abilities, as well as potential divergences with abilities such as beat perception and duration discrimination. These association patterns tempt causal interpretations, such as the hypothesis that good pitch processing is necessary for accurate mistuning perception, and that good mistuning perception is necessary for successful singing performance. However, such correlation patterns always have alternative causal interpretations; for example, one might hypothesize that individual preferences for vocal music drive learning effects for both pitch processing and mistuning perception. A definitive theory of mistuning perception will require these kinds of results to be supplemented by careful experimental studies and causal modeling.

### Author note

For the resources to complete this work and for indefatigable support, we sincerely thank David Poeppel. We are grateful to Klaus Frieler for technical support; to Zaariyah Bashir, Dana Walker, and Oscar Bedford at Goldsmiths and Simone Franz at MPI, for assistance with data collection; to Marcel Zentner for granting permission to use the PROMS; and to Massimo Grassi, who provided access and advice regarding the discrimination tests. P.L.-M. was supported by the Max Planck Society, P.M.C.H. was supported by the EPSRC and the AHRC Centre for Doctoral Training in Media and Arts Technology (EP/L01632X/1), and D.M. was supported by the Humboldt Foundation’s Anneliese Maier research prize.

## Electronic supplementary material

ESM 1 (DOCX 43 kb)

## Footnotes

1 Objective measures of pitch accuracy in melody-accompaniment contexts are lacking in the literature. Nevertheless, we recorded several acoustic measures to ensure that the mistuning of the original versions (inherently linked to the fact that the stimuli were ecologically valid musical material) were in a reasonable range. Further research would be necessary in order to propose valid and reliable pitch accuracy measures in the context of polyphonic music.
