# Supplementary material for: The mistuning perception test: A new measurement instrument
Source: Behav Res Methods. 2019 Mar 28;51(2):663–75. doi: 10.3758/s13428-019-01225-1 (PMC6478636; doi:10.3758/s13428-019-01225-1)
Supplement: Supplementary file 1 — (DOCX 43 kb) [file 13428_2019_1225_MOESM1_ESM.docx]

Table. Reference of the material selected from the *MedleyDB* of Bittner et al. (2014) for the Mistuning Perception Test. Composer, song title and timing selection (start and end of the excerpts) are provided for each of the 37 music pieces used in the construction phase of the test. The greyed rows correspond to the final set of music excerpts (*n* = 30) used in the validation phase. As described in the results section of Experiment 1 (calibration phase), the selection was based on the strength of the relation between the mistuning amount and success rate. Pearson correlation coefficients are reported in the last column.

| **Composer** | **Song title** | **Start - End of the excerpt (min:sec:ms)** | **Correlation between mistuning amount and success rate** |
| --- | --- | --- | --- |
| Alexander Ross | Velvet Curtain | 6:03:900 - 6:15:280 | .78 |
| Ava Luna | Waterduct | 2:08:725 - 2:13:950 |  |
| Brandon Webster | Don't Hear A Thing | 0:09:275 - 0:17:500 | .52 |
| Brandon Webster | Yes Sir I Can Fly | 0:30:850 - 0:37:675 | .59 |
| Clara Berry And Wooldog | Air Traffic | 0:15:020 - 0:24:485 | .79 |
| Clara Berry And Wooldog | Stella | 0:47:050 - 0:52:025 |  |
| Clara Berry And Wooldog | Stella | 1:00:100 - 1:12:120 | .75 |
| Clara Berry And Wooldog | Waltz For My Victims | 0:37:700 - 0:49:850 | .79 |
| Creepoid | Old Tree | 2:08:370 - 2:15:450 | .58 |
| Dreamers Of The Ghetto | Heavy Love | 0:42:000 - 0:52:570 | .64 |
| Faces On Film | Waiting For Ga | 0:23:550 - 0:30:120 | .82 |
| Family Band | Again | 2:06:730 - 2:19:100 | .54 |
| Hezekiah Jones | Borrowed Heart | 0:17:700 - 0:26:680 | .61 |
| Hezekiah Jones | Borrowed Heart | 1:05:700 - 1:17:600 | .78 |
| Invisible Familiars | Disturbing Wildlife | 2:38:250 - 2:44:390 | .55 |
| Liz Nelson | Cold war | 2:35:980 - 2:44:890 | .67 |
| Liz Nelson | Cold war | 0:58:900 - 1:09:650 | .81 |
| Liz Nelson | Rainfall | 0:14:400 - 0:28:800 | .68 |
| Liz Nelson | Rainfall | 1:22:050 - 1:29:450 | .82 |
| Matthew Entwistle | Dont You Ever | 1:42:400 - 1:48:000 |  |
| Music Delta | 80s Rock | 0:25:000 - 0:36:300 | .80 |
| Music Delta | Beatles | 0:24:750 - 0:36:000 | .68 |
| Music Delta | Country1 | 0:25:145 - 0:33:350 | .58 |
| Music Delta | Gospel | 0:52:160 - 1:00:890 |  |
| Music Delta | Reggae | 0:08:650 - 0:17:450 | .59 |
| Music Delta | Rock | 0:08:173 - 0:13:080 |  |
| Music Delta | Rockabilly | 0:16:740 - 0:25:950 | .50 |
| Night Panther | Fire | 0:10:300 - 0:20:300 | .69 |
| Port St Willow | Stay Even | 0:51:420 - 1:00:100 | .66 |
| Secret Mountains | High Horse | 3:20:780 - 3:30:860 |  |
| Secret Mountains | High Horse | 3:09:160 - 3:17:520 | .71 |
| Snowmine | Curfews | 0:52:000 - 1:01:952 | .69 |
| Steven Clark | Bounty | 0:31:050 - 0:40:350 | .81 |
| Strand Of Oaks | Spacestation | 0:32:050 - 0:41:450 | .82 |
| Sweet Lights | You Let Me Down | 1:20:120 - 1:29:205 | .81 |
| The Districts | Vermont | 0:46:800 - 0:58:430 | .77 |
| The Scarlet Brand | Les Fleurs Du Mal | 4:10:440 - 4:16:215 |  |

**Reference**

Bittner, R., Salamon, J., Tierney, M., Mauch, M., Cannam, C., & Bello, J. (2014). MedleyDB: A multitrack dataset for annotation-intensive MIR research. Paper presented at the International Society for Music Information Retrieval (ISMIR), Taipei, Taiwan.
